# Human Three-Dimensional Hepatic Models: Cell Type Variety and Corresponding Applications

**DOI:** 10.3389/fbioe.2021.730008

**Published:** 2021-09-24

**Authors:** Qianqian Xu

**Affiliations:** School of Chinese Medicine, and Centre for Cancer and Inflammation Research, Hong Kong Baptist University, Hong Kong, China

**Keywords:** *in vitro* 3D model, drug development, liver disease, hepatocyte transplantation, hepatic cell types

## Abstract

Owing to retained hepatic phenotypes and functions, human three-dimensional (3D) hepatic models established with diverse hepatic cell types are thought to recoup the gaps in drug development and disease modeling limited by a conventional two-dimensional (2D) cell culture system and species-specific variability in drug metabolizing enzymes and transporters. Primary human hepatocytes, human hepatic cancer cell lines, and human stem cell–derived hepatocyte-like cells are three main hepatic cell types used in current models and exhibit divergent hepatic phenotypes. Primary human hepatocytes derived from healthy hepatic parenchyma resemble *in vivo*–like genetic and metabolic profiling. Human hepatic cancer cell lines are unlimitedly reproducible and tumorigenic. Stem cell–derived hepatocyte-like cells derived from patients are promising to retain the donor’s genetic background. It has been suggested in some studies that unique properties of cell types endue them with benefits in different research fields of *in vitro* 3D modeling paradigm. For instance, the primary human hepatocyte was thought to be the gold standard for hepatotoxicity study, and stem cell–derived hepatocyte-like cells have taken a main role in personalized medicine and regenerative medicine. However, the comprehensive review focuses on the hepatic cell type variety, and corresponding applications in 3D models are sparse. Therefore, this review summarizes the characteristics of different cell types and discusses opportunities of different cell types in drug development, liver disease modeling, and liver transplantation.

## Introduction

The liver is one of the largest organs in the body and plays a critical role in drug metabolism. Hepatic disease accounts for approximately 2 million deaths per year worldwide, of which 1 million are due to complications of cirrhosis and 1 million are due to viral hepatitis and hepatocellular carcinoma (Asrani et al., 2019). Establishing a suitable modeling paradigm is essential for preclinical drug development and disease study. However, species-specific drug metabolizing enzymes and transporters (DMETs) involved in drug absorption, distribution, metabolism, and excretion alter the drug metabolic pathway, hampering the application of animal models in human toxicity prediction (Olson et al., 2000; Cheung and Gonzalez, 2008). Meanwhile, conventional 2D monolayer culture has been proved with uniform exposure to signaling cues and nutrients and less cell–cell and cell–matrix interactions. Thus, rapid dedifferentiation and loss of cellular phenotype were observed in a 2D primary human hepatocyte model, manifesting as a low expression level of key DMETs and decreased albumin production (Rowe et al., 2013). Earliest perturbations on the transcript level in primary hepatocytes were observed after 30 min, and more than 4,000 transcripts were differentially expressed during the first 24 h of culture, significantly affecting pathways involved in the tricarboxylic acid cycle, oxidative phosphorylation, and urea synthesis (Lauschke et al., 2016).

To fill the research gap, development of 3D models that resemble the structure of *in vivo* tissue, imitate cell–cell and cell–matrix interactions, and provide an *in vivo*–like biophysical environment with diverse novel techniques is ongoing. Compared to 2D models, 3D models are promising to replicate morphological and functional features of *in vivo* tissue and retain cellular phenotypes in a relatively long term for repetitive time course measurement and sampling of various endpoints (Bell et al., 2017; Lauschke et al., 2019; Nuciforo and Heim, 2021). Owing to the above, 3D hepatic models show unique benefits in fields of drug development, disease modeling, and liver transplantation. Current breakthroughs on 3D hepatic models include using scaffold-free or scaffold-based culture techniques in the establishment of spheroids, organoids (henceforth defined as an *in vitro* 3D structure which harbors cells with differentiation potential and organ functionality, such as tissue-resident human adult stem cells (hASCs), human embryonic stem cells (hESCs), or human induced pluripotent stem cells (hiPSCs) (Huch and Koo, 2015)), micropatterned co-culture (MPCC) models, and liver-on-a-chip models. Hepatic spheroids are spherical multicellular aggregation which can be generated from one or more hepatic cell types but do not undergo self-organization. The unique spherical structure results in gradient exposure of cells to nutrients, gases, growth factors, and signaling factors from the outside to the center. Therefore, it particularly benefits modeling of spatial zonation of hepatic lobules and the natural architecture of hepatic solid tumor (Cui et al., 2017). Meanwhile, the longevity of this model system is typically limited by the development of a hypoxic and necrotic core with the proliferating cells over time, limiting the diffusion of oxygen into its core (Cox et al., 2020). It was reported that hypoxia would take place in spheroids up to 100–200 μm (Glicklis et al., 2004; Grimes et al., 2014). To create organoids, stem cells are firstly co-differentiated into epithelial and mesenchymal lineages to form spheroids. These spheroids are then embedded in Matrigel and cultured with retinoic acid to further mature. Organoids thus possess self-renewal and self-organization properties that provide a similar composition and architecture to primary tissue and are more suitable than spheroids for investigating long-term processes involving development and degeneration (Huch and Koo, 2015). The MPCC model is established via co-culturing primary human hepatocytes with 3T3-J2 murine embryonic fibroblasts. In contrast to pure PHH monolayers that display a rapid decline in phenotypic functions, this co-culture platform allows interaction between PHH and non-parenchymal cells, maintaining high levels of cytochrome P450 (CYP450) and phase II conjugation enzymes activities for more than 4 weeks (Khetani et al., 2013). The liver-on-a-chip model is created via incorporating microchip fabrication methods into a microfluidic perfusion system. This model contains microchannels that introduce nutrition, oxygen, and signaling cues while removing waste continuously and continuously perfused micrometer-sized cell culture chambers to simulate tissue- or organ-level physicochemical microenvironments. Thus, it is superior in modeling the liver sinusoid, creating a more realistic and dynamic zone-specific culture environment (Gupta et al., 2016). *In vivo*, a hepatic extracellular matrix (ECM) supports structure and signaling trafficking, maintains hepatocyte polarity, and provides the microenvironment for interaction of hepatocyte and immune cells via integrins and other ECM receptors (Treyer and Müsch, 2013; Gissen and Arias, 2015; McQuitty et al., 2020). Owing to its essential role in maintaining hepatic function and disease progression, the ECM should be involved in the establishment of *in vivo*–like 3D models. Scaffold-free techniques are independent of biomaterials imitating the hepatic ECM. Instead, they provide conditions promoting cells to produce their own ECM, which can be achieved via self-aggregation of cells by gravity in hanging drops, culture on an ultra-low attachment surface, large-scale generation by perfused stirred-tank bioreactors, and magnetic levitation of cells preloaded with magnetic nanoparticles. Scaffold-based techniques utilize natural or synthetic external cell anchoring systems that mimic the ECM to facilitate the formation of cell–cell contacts and tissue organization. Common scaffold-based 3D culture paradigms include micropatterned co-culture, microcarrier bead configuration, matrix-embedded, hollow fiber bioreactors, and microfluidics systems (Underhill and Khetani, 2018; Lauschke et al., 2019; Mizoi et al., 2020). Furthermore, 3D bioprinting has been applied as a precise layering method to create scaffolds with a tightly controlled architecture and posit cells or spheroids as building blocks in a specified spatial arrangement necessary for tissue formation (Derakhshanfar et al., 2018; Ma et al., 2018). Compared with scaffold-free techniques, scaffold-based culture configurations have the potential to provide a more delicate biophysical environment for 3D models.

Three hepatic cell types are mainly involved in the above paradigms: primary human hepatocytes isolated from hepatic parenchyma, human hepatic cancer cell lines obtained from hepatocellular carcinoma, and human stem cell–derived hepatocyte-like cells. Different cell types possess unique genetic and protein expression profiles and thus may take specific advantages in divergent research fields. To ensure 3D cell models faithfully recapitulate drug dose response or disease nature, it is essential to select a suitable cell type in the corresponding experiment. Though abundant human 3D hepatic models based on various cell types have been developed, a study that comprehensively summarizes and elaborates this topic is lacking. Therefore, this review is aimed at demonstrating characteristics of different cell types used in current 3D hepatic models and providing guidance for choosing a cell culture system to establish the corresponding 3D model.

## Cell Type Diversity in Human Liver

### Cellular Composition of the Liver

**FIGURE 1:**
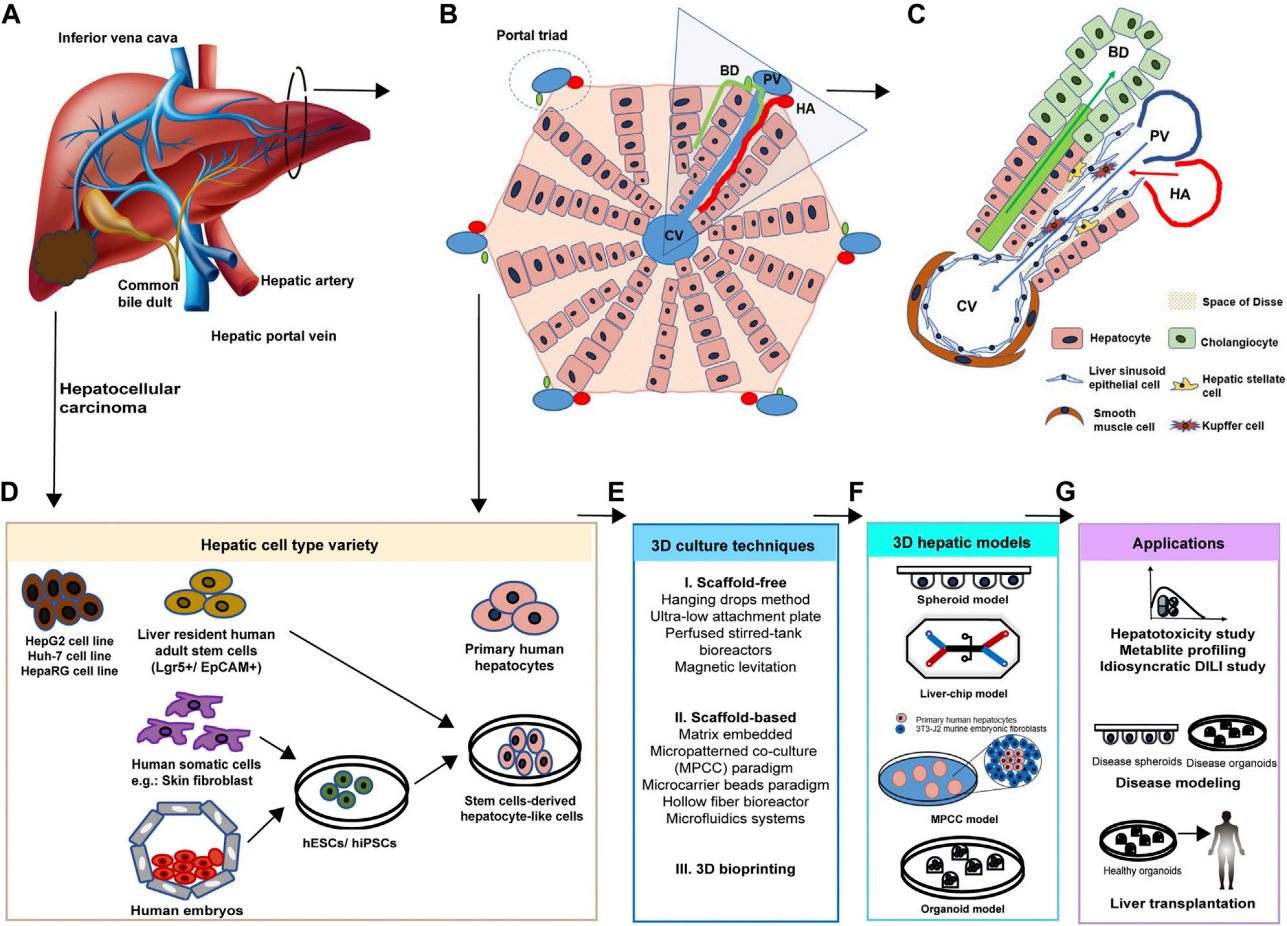
Cellular composition of the liver. **(A)** Gross structure and blood supplies of the liver. The liver is a dark reddish-brown organ supplied by two distinct blood sources: oxygenated blood from the hepatic artery (HA) and nutrient-rich blood from the hepatic portal vein (PV). **(B)** Hepatic lobules are composed of hepatocytes arranged in linear cords radiating out from the central vein (CV) and portal triads including the bile duct (BD), HA, and PV. **(C)** The representative hepatic functional unit in hexagonal hepatic lobules is composed of diverse cell types. Besides parenchymal cells, non-parenchymal cells also support the hepatic structure and function. Hepatic blood vessels are lined by specialized fenestrated liver sinusoidal endothelial cells (LSECs). Kupffer cells (KCs) are macrophages found in the sinusoids. Hepatic stellate cells (HSCs) locate in the space of Disse. Cholangiocytes line the bile ducts. **(D)** Hepatic cell types used in *in vitro* 3D models can be obtained from healthy hepatic tissue, hepatocellular carcinoma, induced somatic cells, and human embryos. **(E)** Advanced 3D culture techniques. Scaffold-free, scaffold-based, and 3D bioprinting techniques are applied to build 3D models. **(F)** Current 3D hepatic models include spheroid, liver-on-a-chip, micropatterned co-culture (MPCC), and organoids models. **(G)** Corresponding applications of 3D hepatic models derived by various cell types. Developed diseased or healthy 3D models are promising in the areas of hepatotoxin screening, idiosyncratic drug reaction study, diverse disease modeling, and hepatocyte transplantation.

The liver has two main lobes, and both are made up of eight segments, supplied by two distinct blood sources: oxygenated blood from the hepatic artery and nutrient-rich blood from the hepatic portal vein (Figure 1A). From microscopic anatomy, the segments are composed of a thousand hexagonal hepatic lobules, possessing a unique structure and function. The hepatic lobules consist of portal triads (i.e., portal vein, bile duct, and hepatic artery) and surrounding hepatocytes arranged in linear cords radiating out a central vein (Figure 1B). As parenchymal cells, hepatocytes are responsible for major hepatic functions, including carbohydrate and lipid metabolism, detoxification, protein synthesis, and self-replication. Besides, resulting from the change of oxygen, nutrients, and signaling cues’ gradient along with the portal triad, hepatocytes are of highly heterogeneity, presenting spatial zonation across the hepatic lobule (Pek et al., 2021). Different subpopulations of hepatocytes show a distinct number of chromosomes, expression profiles of RNA and proteins, and metabolic roles, which are relevant to the gradient of signaling proteins, oxygen, and nutrient supply from the portal to the central zone (Kietzmann, 2019). Additionally, diverse hepatic non-parenchymal cells are significant in maintaining the hepatic structure and function as well (Figure 1C). Among them, hepatic stromal cells are hepatic stellate cells (HSCs) involved in extracellular matrix biosynthesis upon liver injury and liver sinusoidal endothelial cells (LSECs) that form the fenestrated lining of the hepatic sinusoid. Other non-parenchymal cells include resident macrophages: Kupffer cells (KCs), T cells, and dendritic cells, and they all contribute to immune defense (Nuciforo and Heim, 2021). These non-parenchymal cells are appealing to be applied in the establishment of a 3D human hepatic model together with hepatic cell types, since the underlying mechanisms of hepatotoxicity and hepatic diseases involve interaction with these cells. Non-parenchymal cells support the microenvironment for maintenance of hepatic cell function and proliferation as well (Takebe et al., 2013; Ardalani et al., 2019).

### Parenchymal Hepatic Cell Types Used in *In Vitro* 3D Model

Based on the knowledge of cellular composition and function of the liver, parenchymal hepatic cell types to be used in human hepatic 3D models can be obtained from healthy hepatic tissue, hepatocellular carcinoma, induced somatic cells, and human embryos (Figure 1D). After *in vitro* maintenance or differentiation, primary human hepatocytes, human hepatic cancer cell lines (HepG2, Huh-7, and HepaRG cell lines), human adult stem cell–derived HLCs (hASC-HLCs), and human pluripotent stem cell–derived HLCs (hESC-HLCs and hiPSC-HLCs) will be applied in the establishment of spheroid, liver-on-a-chip, MPCC, and organoid models with a range of advanced 3D culture techniques (Figures 1E,F). These models are promising in research areas of drug development, liver disease modeling, and hepatocyte transplantation (Figure 1G).

#### Primary Human Hepatocytes

Obtained from digesting human hepatic parenchyma (Figure 1D), the primary human hepatocyte (PHH) has been considered the golden standard cell type used in the hepatotoxicity study owing to its *in vivo*–like expression of DMETs (Gómez-Lechón et al., 2014). The PHH spheroid model expresses increased activity of phase I metabolism enzymes, including CYP3A4, CYP2C9, CYP2C8, CYP1A2, CYP2D6, CYP2B6, and CYP2C19, in comparison with HepG2 and HepaRG spheroid models, and maintains CYP1A1, CYP2D6, and CYP3A4 activities for at least 35 days (Bell et al., 2016; Berger et al., 2016; Vorrink et al., 2017). Also, the PHH obtained from the patient’s sample can retain patient-specific expression of drug metabolizing enzymes and transporters for investigating patient-specific toxicities (Cox et al., 2020). However, the number and breadth of PHHs are limited by donor availability and invasive procedure to source. Though commercial PHHs have been provided by some companies, variability between batches has been well-recognized.

Previously, maintaining hepatic function and promoting propagation were main challenges to apply PHHs in establishing functional *in vitro* models. Freshly isolated PHHs undergo rapid dedifferentiation with decreased DMET expression and show low expansion capacity when cultured in 2D models (Rowe et al., 2013; Lauschke et al., 2016). This dedifferentiation was linked with reduced activity for transcription factors involved in hepatocyte differentiation, such as hepatocyte nuclear factors 4a and 1a (Oliva-Vilarnau et al., 2020). It was demonstrated that PHHs need unique culture conditions to propagate and maintain mature function *in vitro*. Exogenous supplements need to be added to facilitate the expansion, such as the epidermal growth factor (EGF), tumor necrosis factor (TNF), hepatocyte growth factor (HGF), WNT agonist, and transforming growth factor β (TGF-β) inhibitor (Hu et al., 2018; Xiang et al., 2019). 3D culture paradigms also provide a platform for ECM–cell interactions and cell–cell interactions which help maintain the mature hepatocyte phenotype (Oliva-Vilarnau et al., 2020).

#### Human Hepatic Cancer Cell Lines: HepG2, Huh-7, and HepaRG

Human hepatic cancer cell lines are obtained from human hepatocellular carcinoma (HCC) (Figure 1B). Compared with healthy *in vivo* hepatocytes or PHHs, tumor cell lines were thought to possess a different expression of DMETs due to unique epigenetic regulation and thus may be more suitable for establishing a cancer model instead of the drug screening model (Ingelman-Sundberg et al., 2013; Peng and Zhong, 2015; Nwosu et al., 2018). However, when compared with PHHs, cancer cell lines are superior in unlimited sources, reproducibility, batch-to-batch consistency, and cost-saving.

Commonly used hepatic cancer cell lines include HepG2, Huh-7, and HepaRG cell lines. Similar to the intra-tumor heterogeneity of HCC, the cell lines obtained from different parts or stages of tumor possess distinct cellular phenotypes and metabolic activities as well. HepG2 and Huh-7 were derived from well-differentiated human HCC that still possesses epithelial features and represents HCC in the early stage. HepG2 cells have been shown to maintain secretion of alpha-1 antitrypsin (A1AT), albumin, α-1-acid glycoprotein, alpha-fetoprotein, transferrin, fibrinogen, and plasminogen, though they closely resemble fetal hepatocytes instead of adult hepatocytes (Rowe et al., 2013; Cox et al., 2020). Compared with the Huh-7 cell line, the HepG2 cell line possesses wild-type *p53*, lower chromosome numbers, and lower proliferation (41–57 h versus 23–27 h doubling time), exhibiting human epigenetic chromatin modification enzymes like PHHs (Nwosu et al., 2018; Ruoß et al., 2019; Rodríguez-Hernández et al., 2020).

HepaRG is the bipotent progenitor cell line derived from a chronic hepatitis C–related HCC (Gripon et al., 2002), possessing relatively slow mitotic activities (48–69 h). Interestingly, HepaRG cell lines can be differentiated to a mixture of hepatocyte islands and cholangiocyte-like cells upon exposure to dimethylsulfoxide (DMSO) or forskolin. The stable expression of CYP3A4, CYP1A2, drug transporters, and transcription factors was noted in differentiated HepaRG cells after treatment with DMSO (Aninat et al., 2006; Andersson et al., 2012; Rubin et al., 2015; Mayati et al., 2018). In 2D monolayer culture, HepaRG shows a higher expression of key DMETs more closely resembling PHHs as compared to HepG2 cell lines (Sison-Young et al., 2015). However, due to the longer time required for expansion and differentiation, HepaRG was less incorporated into high-throughput analyses (Cox et al., 2020).

#### Human Stem Cell–Derived Hepatocyte-Like Cells: Human Adult Stem Cell–Derived Hepatocyte-Like Cells, Human Induced Pluripotent Stem Cell–Derived Hepatocyte-Like Cells, and Human Embryonic Stem Cell–Derived Hepatocyte-Like Cells

Human stem cell–derived hepatocyte-like cells (HLCs) can be induced from either liver tissue–resident human adult stem cells (hASCs) or pluripotent stem cells, hESCs and hiPSCs. Stem cells are capable of self-renewing and differentiating into multiple lineages. Hence, they provide unlimited sources to generate patient-specific HLCs, which exhibit mature hepatic function under special culture conditions (Calabrese et al., 2019; Carpentier et al., 2014; Huch et al., 2015; Huch and Koo, 2015; Takayama et al., 2012).

Reported hepatic hASCs are *Lgr5* ^+^ (leucine-rich repeat–containing G-protein–coupled receptor 5^+^) and EpCAM^+^ (epithelial cell adhesion molecule–expressing^+^) liver progenitor cells, and they can be obtained from the patients’ liver tissue sample. The *Lgr5* gene has been taken as a marker of E9.5–E10.0 bipotent liver progenitors residing at the apex of a liver hepatoblast hierarchy, and upon liver damage, *Lgr5* becomes highly upregulated in a subset of cells, contributing to the regeneration of the tissue (Huch et al., 2015; Prior et al., 2019). Epithelial cell adhesion molecule–expressing (EpCAM^+^) cells also display characteristics of liver progenitors (i.e., self-renewal and bipotency) as proven by clonogenic and differentiation assays (Schmelzer et al., 2007; Dorrell et al., 2014; Tarlow et al., 2014). To date, hESCs can be obtained either from embryos at morula and blastocyst stages or purchasing commercial cell lines including commonly used HSF6, HUES7, and H9 hESCs, while hiPSCs can be induced through reprogramming diverse human somatic cell types including liver tissue. Compared to hESCs, hiPSCs are available resources and maintain the patient’s specific genetic changes, which benefits personalized treatment. Skin fibroblasts are the most recommended to use since they are easily isolated from skin biopsies (Takahashi and Yamanaka, 2006).

To generate hiPSCs, reprogramming factors can be integrated into somatic cell genomes via single-cassette vectors (Cre-Lox), mRNA or miRNA transfection, episomal plasmids, or non-integrating viral vectors (Takahashi et al., 2007; Raab et al., 2014; Ramboer et al., 2015; Mitani et al., 2017; Ang et al., 2018; Dao Thi et al., 2020). Furthermore, to characterize derived hESCs and hiPSCs, the expression of stage-specific embryonic antigen 4 (SSEA-4) and transcription factors OCT-3/4 and NANOG has been identified to verify the pluripotency (Allegrucci and Young, 2007; Carpentier et al., 2014). The idea of inducing hASCs, hESCs, and hiPSCs into HLCs is based on the natural development of the liver, when pluripotent epiblast cells sequentially turn into primitive streak, definitive endoderm, posterior foregut endoderm, embryonic liver progenitors, and hepatocytes under a series of stimulations (Ang et al., 2018; Ober and Lemaigre, 2018; Prior et al., 2019). To generate human adult stem cell–derived hepatocyte-like cells (hASC-HLCs), after liver biopsy, cell isolation, and selection, hASCs can be cultured with a combination of growth factors including HGF, EGF, fibroblast growth factor (FGF), WNT agonist, TGF-β, and Rspo1 to induce activated bipotent liver progenitors that are capable of self-organization. Furthermore, blockade of ductal fate by the NOTCH inhibitor, in combination with the TGF-β signaling inhibitor, removal of Rspo1, and addition of dexamethasone and bone morphogenetic protein (BMP) are applied to facilitate the differentiation of bipotent liver progenitors toward hASC-HLCs (Lemaigre, 2009; Zong et al., 2009; Huch et al., 2015). hASC-HLCs have been proved with hepatic function such as cytochrome activity and albumin, A1AT, and bile acid production *in vitro* (Huch et al., 2015). Similarly, current protocols to differentiate hESCs and hiPSCs toward HLCs usually involve treatment with 1) activin A and/or TGF-β to obtain definitive endoderm cells; 2) FGF-2 and BMP-4 to induce liver progenitors; and 3) HGF, oncostatin M (OSM), dexamethasone, and/or co-culture with other cell types to specify hepatocytes. Differentiated hESC-HLCs and hiPSC-HLCs were proved to express hepatic cellular markers including tyrosine aminotransferase, A1AT, and CYP7A1 and exhibit hepatic functions such as inducible CYP450 activity, albumin secretion, urea synthesis, glycogen storage, and low-density lipoprotein (LDL) uptake (Cai et al., 2007; Carpentier et al., 2014; Touboul et al., 2010). Transcriptional changes and immunocytochemistry revealed that differentiated hESC-HLCs showed more similarity to pericentral rather than periportal hepatocytes by exhibiting a pericentral hepatocyte marker, glutamine synthase (Baxter et al., 2015).

In addition to separated induction and differentiation procedures illustrated above, induced HLCs can be generated using direct lineage reprogramming technology which converted human fibroblasts to functional hepatocytes through overexpression of lineage-specific transcription factors (Vierbuchen and Wernig, 2012; Du et al., 2014; Huang et al., 2014). It has been proposed that, during lineage reprogramming, one cell type can be converted directly to the final mature state of another cell type bypassing its intermediate states. Huang et al. reported the application of lentiviruses carrying human pioneer factor *FOXA3*, together with liver-enriched transcription factors *HNF1A* and *HNF4A*, successfully induced conversion from human fibroblasts into HLCs, which exhibited mature hepatic functions comparable to cryopreserved PHHs instead of hepatic progenitor cells, including CYP450 enzyme activities and biliary excretion of drug compounds. Further genome-wide expression profile analysis and gene set enrichment analysis indicated that human fibroblasts underwent hepatic conversion by transcriptional alterations at the whole-genome level. (Huang et al., 2014). Du et al. reported viral-mediated overexpression of transcription factors *HNF1A*, *HNF4A*, and *HNF6* along with maturation factor *PROX1* and liver-enriched transcription factors *ATF5* and *CEBPA* successfully induced conversion from human fibroblasts into HLCs, which possessed metabolic activities of CYP3A4, CYP1A2, CYP2B6, CYP2C9, and CYP2C19 comparable to fresh PHHs.

Nevertheless, one should be cautious when considering the actual maturity of HLCs. Further maturation after differentiation of hiPSC/hESC-HLCs was proposed due to fetal-like hepatic characteristics of HLCs, such as drug metabolism capacity, albumin secretion level, and urea secretion level, which are considered lower than those of fresh adult PHHs (Takayama et al., 2012; Baxter et al., 2015). The previous study indicated that the average and variance of CYP3A4 activity levels in PHH-derived hiPSC-HLCs, non-PHH–derived hiPSC-HLCs, and hESC-HLCs were similar to each other, but the CYP1A2, CYP2C9, and CYP3A4 activity levels in the PHH-hiPSC-HLCs were estimated to be around 60% of those of their parental PHHs (Takayama et al., 2014). The albumin and urea secretion levels in HUES7 cell–derived hESC-HLCs were approximately 8-fold and 18-fold lower than those from fresh adult PHHs, respectively. Meanwhile, principal component analysis revealed that differentiated hESC-HLCs and hiPSC-HLCs possessed a high expression level of alpha-fetoprotein, glutathione S-transferase π, and heat shock protein 47 and a low level of CYP2A6 and ADH activity, which were comparable to those of fetal PHHs instead of adult PHHs (Rowe et al., 2013; Baxter et al., 2015). It has been suggested that the 3D culture technique with a natural or synthetic ECM support or cell–cell contact can promote the maturation of the hiPSC/hESC-HLCs and maintenance of hepatic function. When compared with a monolayer culture model, these hiPSC/hESC-HLCs’ 3D model exhibited a higher expression level of hepatic-specific gene and superior capability in adult hepatic function (Nagamoto et al., 2012; Ramasamy et al., 2013; Takayama et al., 2013).

Collectively, the sources and divergent characteristics of the above three cell types are summarized in Table 1. These unique properties of cell types indicate their advantages in different research fields of *in vitro* 3D modeling paradigm as discussed in the below part.

**TABLE 1:** Summary of characteristics of cell types used in human 3D hepatic models.

Cell type	Source	Feature
PHHs	Fresh or cryopreserved healthy human liver tissue	Limited proliferative capacity *in vitro*
		Batch-specific property
		Possessing mature hepatocyte’s function
hASC-HLCs	Human liver progenitor cells	Preserved donor’s genetic background
		Possessing hepatocyte’s function
hESC-HLCs	Human embryos at morula or blastocyst stage	Possessing fetal-like hepatocyte maturity
		Less age-related genetic change
		Showed more similarity to pericentral hepatocytes
hiPSC-HLCs	Reprogrammed human somatic cells	Preserved donor’s genetic background
		Unlimited resources
		Possessing fetal-like hepatocyte maturity
HepG2 cell line	Well-differentiated human HCC	Unlimited proliferation
		Tumorigenic
		More resembling fetal hepatocytes
Huh-7 cell line		Unlimited proliferation
		Tumorigenic
		Impaired hepatocyte’s function
		Possessing more tumor phenotypes
HepaRG cell line	Chronic hepatitis C–induced human HCC	Unlimited proliferation and tumorigenic
		More resembling PHH functions than HepG2 and Huh-7 cell lines
		Possessing properties of hepatic progenitors

PHHs, primary human hepatocytes; hASCs, human adult stem cells; hiPSCs, human induced pluripotent stem cells; hESCs, human embryonic stem cells; HLCs, hepatocyte-like cells; HCC, hepatocellular carcinoma.

## Hepatic Cell Types and Corresponding Application With 3D Cell Models

### Drug Development

#### Hepatotoxin Screening to Avoid Drug-Induced Liver Injury

A life-threatening adverse drug reaction, drug-induced liver injury (DILI), is accompanied by oxidative stress, metabolite-induced hepatotoxicity, and activated innate and adaptive immune responses (Donato and Tolosa, 2021). Of the affected patients, 9.4% die or require liver transplantation and 18.9% show persistent liver damage 6 months after DILI diagnosis (Fontana et al., 2014). In addition to clinical importance, DILI is responsible for the most post-marketing withdrawals of drugs. In the last 30 years, 14 drugs have been withdrawn from the US and European markets due to hepatotoxicity shown in post-marketing stages, representing a financial burden for the pharmaceutical industry (Zhou et al., 2019). One of the reasons for high incidence of DILI is an unsuitable preclinical hepatotoxin screening and assessment model, as short-term 2D cell models usually lead to incompetent drug metabolism and restrict the predictivity of DILI. To fill this gap, more predictive *in vitro* models need to be developed for preclinical drug screening.

The current hepatic 3D model for DILI prediction mainly utilized PHH cell type (Table 2). Khetani et al. established the PHH MPCC model to evaluate the hepatotoxicity of 35 DILI-positive and 10 DILI-negative compounds listed by Xu and colleagues (Xu et al., 2008), along with albumin, urea, ATP, and glutathione (GSH) levels as the endpoints for DILI identification. Upon four repeated dosages over the time course of 9 days, the MPCC model correctly predicted 23 out of 35 DILI-positive compounds (65.7% sensitivity) with one out of ten false-positive compounds (90% specificity) (Khetani et al., 2013). For chronic hepatotoxicity assays, Bell and colleagues proved that preserved hepatic phenotypes and long-term functionality in the PHH spheroid models allowed them to be applied. They dosed PHH spheroids every 2 days with five hepatotoxins: amiodarone, bosentan, diclofenac, fialuridine, and tolcapone, and determined the cell viability via detecting cellular ATP content after 48 h and 8 and 28 days. Significant reduction in the EC_50_ values for all tested compounds was observed between 48 h and 8 days, reflecting toxicity *in vitro* at clinically relevant concentrations. Long-term cytotoxicity was most prominent for fialuridine, for which cytotoxicity was detected as 0.1 μM at 28 days compared with EC_50_ > 100 μM at 48 h, highlighting the essential role of the PHH 3D culture model in long-term hepatocytotoxicity screening (Bell et al., 2016). Meanwhile, three larger panels of hepatotoxicity studies have been conducted in PHH spheroids to date with ATP quantifications as the single endpoint (Proctor et al., 2017; Vorrink et al., 2018; Li et al., 2020). Proctor et al. used high-throughput 3D InSight™ Human Liver Microtissues spheroid models co-culturing PHHs, KCs, and LSECs to test the hepatotoxicity of 110 drugs (69 DILI-positive and 41 DILI-negative). PHH co-culture spheroids after a 14-day repeated exposure predicted DILI with 19–61% sensitivity and 81–98% specificity, depending on thresholds employed (Proctor et al., 2017). In contrast, Li et al. established PHH mono-culture spheroids to test the hepatotoxicity of 100 drugs (62 DILI-positive and 38 DILI-negative). Upon 14 days of repeated exposure, the model reported prediction of DILI with 32–61% sensitivity and 79–95% specificity, depending on thresholds employed (Li et al., 2020). Interestingly, though for above PHH-derived models, whether co-culture with non-parenchymal cells or not seems to not significantly affect the differentiation between DILI-positive and -negative drugs, KCs were proved to potentiate the cytotoxicity induced by trovafloxacin and acetaminophen in PHH/KC co-culture spheroids, manifested as a lower IC_50_ value when compared with PHH mono-culture. However, a protective role of activated KCs in co-culture compared to mono-culture was shown if co-treated with acetaminophen and lipopolysaccharides. Additional tests with 14 DILI-positive compounds comparing mono-culture and co-culture spheroids indicated that KCs exerted compound-dependent differential effects on drug-induced cytotoxicity (Li et al., 2020). Vorrink and colleagues got better screening outcomes with higher sensitivity via establishing PHH mono-culture spheroids in specific chemically defined conditions which were demonstrated to retain PHH viability, functionality, and transcriptomic, proteomic, and metabolomic phenotypes for multiple weeks (Bell et al., 2016; Bell et al., 2017; Vorrink et al., 2017). Upon repeated exposure to 123 drugs (70 DILI-positive and 53 DILI-negative) for 14 days, PHH spheroids predicted the hepatotoxicity of 48 out of 70 compounds (sensitivity = 69%), and no false-positive result was reported (specificity = 100%). Furthermore, this model correctly distinguished five pairs of DILI-positive drugs (amodiaquine, troglitazone, clozapine, nefazodone, and bosentan) and their DILI-negative structural analogs (primaquine, rosiglitazone, amoxapine, buspirone, and ambrisentan), underlining the potential of PHH-derived 3D models in evaluating drug candidates at chemical derivatization stages during drug development (Vorrink et al., 2018).

**TABLE 2:** Selected hepatotoxin screening using 3D hepatic models established with different cell types.

Cell type	Culture paradigm	Drug exposure period	Endpoints	References
PHHs	MPCC co-culture	9 days	Albumin, urea, and glutathione levels and cellular ATP content	Khetani et al. (2013)
PHHs	Spheroid co-culture	14 days	Cellular ATP content	Proctor et al. (2017)
PHHs	Spheroid mono-culture	14 days	Cellular ATP content	Li et al. (2020)
PHHs	Spheroid mono-culture	14 days	Cellular ATP content	Vorrink et al. (2018)
PHHs	Spheroid mono-culture	28 days	Cellular ATP content	Bell et al. (2016)
PHHs	MPCC co-culture	14 days	Cell viability	Ware et al. (2017)
			Albumin secretion	
			Urea synthesis	
			CYP3A4 activity	
			Global gene expression profiles	
HepG2	Spheroid mono-culture	6 days	Oxidative stress response pathway	Hiemstra et al. (2019)
			UPR pathway	
			DNA damage response pathway	
HepaRG	Spheroid mono-culture	6 days	Cellular ATP content	Ramaiahgari et al. (2017)
HepaRG	Spheroid mono-culture	7 days	Cellular ATP content	Ott et al. (2017)
			CYP1A and CYP3A4 activity	
hiPSC-HLCs; hESC-HLCs; HepG2	Spheroid mono-culture	24 h	Cell viability	Takayama et al. (2013)
hiPSC-HLCs; hESC-HLCs	Organoid mono-lineage	72 h	Cell viability	Shinozawa et al. (2021)
			Cholestatic function	
			Mitochondrial toxicity	

PHHs, primary human hepatocytes; MPCC, micropatterned co-culture; hESCs, human embryonic stem cells; hiPSCs, human induced pluripotent stem cells; HLCs, hepatocyte-like cells.

Nevertheless, merely adopting functional endpoints as parameters failed to elucidate detailed mechanistic insights into diverse pathways involved in liver hepatotoxicity. Instead, global gene expression profiling has the potential to reveal the mechanism of action of hepatotoxins by identifying genes that are candidate biomarkers of adverse drug effects. Meanwhile, it seems more sensitive in hepatotoxic identification than conventional cellular functional endpoints (Barros and Martin, 2008; Ware et al., 2017). Ware and colleagues established PHH MPCC models and confirmed global gene expression patterns in these models can be used to distinguish hepatotoxic drugs (troglitazone, nefazodone, ibufenac, and tolcapone) from their non-toxic analogs (rosiglitazone, buspirone, ibuprofen, and entacapone). They treated MPCC models with pair drugs at their respective non-toxic Cmax for 1, 7, and 14 days and then assessed the functional endpoints (cell viability, albumin secretion, urea synthesis, and CYP3A4 activity) and global gene expression profiles via Affymetrix whole genome human microarrays at each time point. Interestingly, at a non-toxic dose of both toxins and non-toxins, PHH viability, hepatic urea secretion, and albumin level were not significantly affected over 2 weeks, while different transcript perturbations involved in KEGG pathways and GO processes were noted with time change, suggesting that hepatotoxins led to a greater number of differentially expressed transcripts than corresponding non-toxic analogs (Ware et al., 2017). Except for gene expression profiling, cellular labeling technique inserting exogenous reporter genes into hepatic cell genomes can be combined with a 3D culture paradigm to monitor DILI-relative pathways such as oxidative stress response pathways, unfolded protein response pathway, and DNA damage response pathway, so as to explore the drug-specific DILI mechanism (Wink et al., 2018; Hiemstra et al., 2019). Hiemstra et al. established spheroid models by using six HepG2-GFP cellular stress reporter cell lines representing Nrf2 activation (Srxn1-GFP and NQO1-GFP), unfolded protein response (BiP-GFP and Chop-GFP), and DNA damage response (p21-GFP and Btg2-GFP). Upon 6 days of daily repeated exposure with 33 compounds (20 most-DILI-concern drugs, 7 less-DILI-concern drugs, and 6 no-DILI-concern drugs), strongest stress response activation was observed at the sixth day in HepG2-GFP spheroids, with the BiP and Srxn1 pathways being most responsive for most-DILI-concern compounds, indicating cellular stress reporter cell line–derived spheroid models were promising for mechanism-based identification of compounds with liability for DILI (Hiemstra et al., 2019).

Except for PHH cell type, HepaRG cell type was also used in some small panels of drug testing. Ramaiahgari et al. established the HepaRG spheroid with retained functionality for 28 days. Upon repeated exposure to two pairs of hepatotoxins (trovafloxacin, troglitazone) and non-toxic analogs (levofloxacin, rosiglitazone) for 6 days, dose-dependent cytotoxicity with hepatotoxins compared with their non-toxic analogs was observed in HepaRG spheroids. Besides, the model correctly identified DILI-positive compounds: acetaminophen, diclofenac, isoniazid, and cyclosporine A (Ramaiahgari et al., 2017). Similarly, Ott et al. developed HepaRG spheroids along with multiple cellular functional endpoints. Upon repeatedly exposing spheroids to 10 hepatotoxins and 2 non-toxins for 7 days, the model correctly flagged 7/10 compounds as hepatotoxins with 100% specificity (Ott et al., 2017).

However, sensitivity of HepG2 and HepaRG cell types to hepatotoxins was still considerably lower than that in PHH models which had been seen as the gold standard cell type for predicting human hepatotoxic drugs (Gómez-Lechón et al., 2014; Hendriks et al., 2016; Zhou et al., 2019). Hendriks et al. compared the sensitivity of PHH spheroids and HepaG2 spheroids to acetaminophen- and tetracycline-induced hepatotoxicity along with albumin secretion as the endpoint. Upon 8 days of repeated exposure, the PHH spheroid exhibited a lower TC_50_ (1.3 mM) than the HepaRG spheroid (1.8 mM) of acetaminophen and lower TC_50_ (0.1 mM) than the HepaRG spheroid (0.2 mM) of tetracycline (Hendriks et al., 2016). Furthermore, Zhou et al. comparatively reviewed the sensitivity of current hepatic 3D models to acetaminophen-induced hepatotoxicity. Under the same drug exposure and mono-culture condition, the PHH-derived spheroid exhibited a lower TC_50_ than the HepaRG spheroid, while the HepaRG spheroid exhibited a lower TC_50_ than the hiPSC-HLC spheroid and HepG2 spheroid (Zhou et al., 2019). Though this comparison only focused on the spheroid model and acetaminophen-induced hepatotoxicity, it confirmed the superior PHH cell type in hepatotoxicity studies, which has been proved from the perspective of genetic profiling and expression as well. In spheroids, PHHs showed that proteomic signatures closely resemble the human liver *in vivo* and retained their transcriptomic and metabolomic profiles for multiple weeks (Bell et al., 2016; Bell et al., 2017; Vorrink et al., 2017; Bell et al., 2018). In contrast, transcriptomic patterns of other cell types differed substantially. 8,148 out of 17,462 genes analyzed were differentially expressed in PHH spheroids compared to HepaRG cells (Bell et al., 2017).

For DILI study using stem cell–derived hepatocyte-like cells, Takayama et al. established hiPSC-HLCs spheroids to compare their susceptibility toward drug-induced hepatotoxicity with that of HepG2 spheroids. hiPSC-HLC spheroids showed higher susceptibility along with less cell viability in the treatment with 19/22 hepatotoxins for 24 h. Meanwhile, hepatotoxins caused cell death in hiPSC-HLC spheroids, which was partially rescued by treatment with CYP inhibitors, suggesting that hiPSC-HLC spheroids are promising in screening toxicity of the reactive metabolites that were metabolized by CYP enzymes. Additionally, they also compared the maturation of hepatic function between monolayer models and spheroid models. When compared with hESC-HLC mono-culture, hESC-HLC spheroids showed a higher gene expression level of albumin, CYP enzymes, conjugation enzymes, hepatic transporters, hepatic nuclear receptors, hepatic transcription factors, and bile canalicular transporters. The ability of bile acid uptake and efflux and secretion level of albumin and urea in the spheroid model were also superior to those in the monolayer model. When compared with the hiPSC-HLC monolayer culture model, hiPSC-HLC spheroids exhibited higher CYP induction potency and CYP2C9 and CYP3A4 activity levels after treatment with rifampicin for 48 h (Takayama et al., 2013). Moreover, Shinozawa and colleagues developed the hiPSC/hESC-HLC organoid model with bile transport function, which allowed for measurement of cell viability and cholestatic and mitochondrial toxicity (Shinozawa et al., 2021). Upon exposure to 238 marketed drugs, the model reported 88.7% sensitivity and 88.9% specificity, which provided values comparable to or higher than those in previous studies with PHH models (Xu et al., 2008; Khetani et al., 2013; Proctor et al., 2017; Vorrink et al., 2018). With proved bile transport function, this model supported mechanism classification of DILI, including cholestatic toxicity, mitochondrial toxicity, bile salt export pump deficit, or further unknown vulnerable mechanism. Different from the simplified culture model without hepatic canaliculi structure, organoid models established with directed hiPSCs/hESCs formed the unidirectional bile acid transport pathway, which faithfully mimicked the metabolite excretion into bile canaliculi *in vivo.* Meanwhile*,* compared with the reported PHH sandwich culture, it is believed the pluripotent stem cell resource could solve reproducibility and high-throughput challenges (Shinozawa et al., 2021).

Taken together, it has been suggested that repeated exposure for at least 6 days came with higher sensitivity in comparison with 24 or 48 h single exposure (Ramaiahgari et al., 2014; Ott et al., 2017; Proctor et al., 2017; Ramaiahgari et al., 2017; Hiemstra et al., 2019; Li et al., 2020). And though there are diverse measuring approaches for detecting DILI-relevant cytotoxicity, taking the cellular ATP level as the single endpoint was sufficient for distinguishing between hepatotoxins and non-toxins, which fits the large screening needs of early drug discovery and compound structure design based on structural toxicity (Shah et al., 2015). Though the PHH model is still a mainstream screening platform with superior maturation of hepatic function, 3D culture models which support ECM formation and cell–cell contact can promote further maturation of stem cell–derived HLCs. hESC/hiPSC-HLC 3D models, especially organoid models, have shown comparable predictive power and unique advantages in imitating *in vivo* tissue–like structures and cellular composition, including bile canaliculi, zone-specific architecture, hepatic mesenchyme, endothelial cells, and cholangiocytes (Shinozawa et al., 2021). Therefore, they are promising in cholestatic, hepatocellular, or further unknown complex DILI mechanism studies. It is believed that stem cell–derived HLCs would serve as an alternative hepatotoxin screening platform and overcome current resource shortage and reproduction challenge when using PHHs.

#### Idiosyncratic Drug Reaction

Except for routine hepatotoxicity screening to avoid DILI for most patients, the idiosyncratic drug reaction study focusing on the influence of genetic polymorphisms is significant for clinical prescription as well. Although the most DILI-related acute liver failure was dose-dependent, roughly 10–15% of it can be attributed to individual effects instead of dose-caused effects (Reuben et al., 2010). Genetic polymorphism, especially single-nucleotide polymorphism (SNP), is related to interindividual differences in CYP metabolism capacity and immune-mediated drug responsiveness (Ingelman-Sundberg, 2001; Gerussi et al., 2021). Current approaches for idiosyncratic drug response are relatively limited since routine hepatotoxicity screening mostly relies on PHH cell type obtained from healthy livers with fully functional DMETs, which may not be able to reveal the risk of genetic polymorphisms. Thus, cell systems with corresponding defects and a novel strategy to reveal the relationship of genotype and idiosyncratic drug reaction are needed (Chalasani and Björnsson, 2010; Urban et al., 2014; Roth and Lee, 2017). The establishment of human hepatic 3D models with genetic and phenotypic variability in pharmacokinetics provides insight into the pathogenesis of idiosyncratic drug effects.

Among various CYPs expressed in the liver, CYP2D6 handles a quarter of commercially used drugs and has been proved with diverse phenotypic variability owing to single-nucleotide polymorphism (SNP) (S. F. Zhou, 2009). Vorrink and colleagues utilized patients’ PHH spheroids to evaluate the impact of genetic polymorphism on the metabolic fate of dextromethorphan. *In vivo*, dextromethorphan is primarily metabolized to dextrorphan by CYP2D6; in case of CYP2D6 deficit, it can be metabolized to 3-methoxymorphinan by CYP3A4. In this study, PHH spheroids with genotypically defined extensive (CYP2D6 *1/*1 and CYP2D6 *1/*4) and poor (CYP2D6 *4/*10) CYP2D6 enzymes were exposed to dextromethorphan. The poor metabolizer showed shunted metabolic flux toward CYP3A4-dependent metabolism, resulting in the predominant formation of 3-methoxymorphinan (Vorrink et al., 2017). This outcome was consistent with *in vivo* dextromethorphan metabolism, suggesting that genetic polymorphism–related drug response can be presented in PHH spheroids (Vorrink et al., 2017). In addition to PHH models, *in vitro* models established with pluripotent stem cells (HLCs) from different donors are promising for exploring individual susceptibility as well. Importantly, hiPSCs can be reprogrammed from different somatic cell types and are highly reproducible, which solves the challenge of PHH shortage. To prove that the variable genetic background is also retained in the derived HLCs, Takayama et al. generated HLCs from 12 donors’ PHHs with various SNPs on CYP2D6. By comparing PHH-hiPSC-HLCs and parental PHHs in CYP2D6 enzyme metabolism capacity, expression of CYP genes and expression of SNPs on CYP genes, and responses to 72-h treatment with CYP2D6-activated drug tamoxifen and responses to 24-h treatment with CYP2D6-detoxified drug desipramine and perhexiline, they proved that interindividual differences in CYP2D6-mediated metabolism and hepatotoxicity due to SNPs in *CYP2D6* were reproduced in the PHH-hiPSC-HLCs (Takayama et al., 2014). Therefore, *CYP2D6* polymorphism–mediated drug-induced hepatotoxicity could also be predicted in hiPSC-HLC culture models. The CYP variant CYP2C9*2 is also known for encoding proteins with reduced enzymatic activity in comparison with wild-type CYP2C9*1 (Rettie et al., 1994)*.* Shinozawa et al. established an hiPSC-HLC organoid model from eight donors with CYP2C9*2 enzyme activity as normal (*C/C*) or intermediate (*C/T*) to study genomic predisposition to bosentan-induced cholestasis. They found that, in *C/T* allele carrier organoids, the excretion of fluorescent bile acid into organoids was severely impaired by bosentan (positive rate: *C/C*, 17.1% positive; *C/T*, 70.8% positive) (Shinozawa et al., 2021). This result was in accordance with the CYP2C9*2 polymorphism–related bosentan-induced DILI study in patients (Markova et al., 2013).

The idiosyncratic drug reaction screening research can be independent of identifying CYP polymorphism. For instance, the hiPSC-HLC organoid model was applied to support the DILI-related genome-wide association study (GWAS) and underlying DILI-vulnerable pathway identification. Koido and colleagues established PHH 2D models and hiPSC-HLC organoid models to assess the DILI-predictive power of polygenic risk score (PRS). In this study, whole-genome genotypes of PHHs and hiPSC-HLCs from different healthy donors were determined by an SNP array to calculate their PRSs based on the previous GWAS. Upon exposure of 12 hepatotoxic drugs, the PRS-dependent cytotoxic trend across multiple drugs was noted in both PHH 2D models and hiPSC-HLC organoid models, revealing a shared DILI predisposition which was correlated with polygenic scores but independent of properties of each specific drug. In addition, transcriptome analysis of hiPSC-HLC organoid models proved that expression levels of mitochondrial toxicity–related genes and unfolded protein response–related genes were correlated with the PRS (Koido et al., 2020). Similarly, hASC-HLCs also maintain interindividual variability in drug response and can be adopted as an initial screening platform for an idiosyncratic drug event. Wang et al. (2010) established hASC-HLC spheroids from six donors to reveal idiosyncratic events of eight tyrosine kinase inhibitors (TKIs): erlotinib, lapatinib, cabozantinib, foretinib, gefitinib, crizotinib, ceritinib, and tepotinib. Dose-dependent toxicity curves obtained in these spheroids were significantly different upon 48-h treatment of four drugs: erlotinib (TC_50_ ranged from non-detected to 24.05 ± 10.9 μM), lapatinib (TC_50_ ranged from non-detected to 65.1 ± 56.6 μM), cabozantinib (TC_50_ ranged from non-detected to 36.3 ± 11.9 μM), and foretinib (TC_50_ value ranged from non-detected to 130.5 ± 43.4 μM), suggesting that heterogeneity influenced the toxicity of these four TKIs, while the other four TKIs showed strong toxicity to all spheroids derived from different donors, indicating that their cytotoxicity effects were less affected by individual heterogeneity (Wang et al., 2019).

Collectively, genetic polymorphism–relevant interindividual differences in drug responses were proved to be retained in PHH, hASC-HLC, and hiPSC-HLC culture models, enabling preclinical idiosyncratic drug response screening (Koido et al., 2020; Shinozawa et al., 2021; Takayama et al., 2014; Vorrink et al., 2017; Wang et al., 2019). Particularly, establishing the hiPSC-HLC biobank and applying them in idiosyncratic drug event screening is a promising strategy to overcome the shortage of PHH resources and fully reveal interindividual variations in drug response due to genetic makeup. In the future, dosing these cell types with drugs under different diseased backgrounds such as HBV infection, NAFLD, and HCC should also provide clues to patient-specific drug adverse responses (Lin and Khetani, 2016). Meanwhile, as mentioned above, the idiosyncratic drug reaction research can be independent of identifying CYP polymorphism. Several ongoing research studies have revealed the significance of immunogenetic in idiosyncratic drug response (Gerussi et al., 2021); in the context of this condition, genome-wide analysis and relevant statistic models would provide a new prospective in addition to merely focusing on DMET-related candidate genes, and both PHHs and hiPSC-HLCs are compatible with this technique (Koido et al., 2020).

### Liver Disease Modeling

#### Non-Alcoholic Fatty Liver Disease

Non-alcoholic fatty liver disease (NAFLD) encompasses a series of hepatic parenchymal damage with varying degrees, ranging from simple fat accumulation and non-alcoholic steatohepatitis (NASH) with subsequent inflammation and necrosis, to hepatic fibrosis, cirrhosis, and even HCC. NAFLD typically develops in patients with the metabolic syndrome or ectopic lipid deposition–induced insulin resistance resulting from chronic energy surplus. Insulin resistance induces glucose conversion into adipose tissue in the liver via hepatic *de novo* lipogenesis and disrupts lipolysis, contributing to hepatic inflammation, oxidative stress accumulation, mitochondrial damage, and cell apoptosis (Thiagarajan et al., 2021). Among all disease stages, NASH is thought to be the turning point to the life-threatening stages, as HSCs differentiate into myofibroblast-like cells, which cause fibrosis and predispose patients to cirrhosis and hepatocellular carcinoma in NASH. However, no FDA-approved drug therapy for NASH exists currently, and the conservative treatment depends on lifestyle intervention and weight control, highlighting the need of pathogenesis study and exploration of new drug targets (Boeckmans et al., 2018). 3D cell models of NAFLD can be induced via exposure of appropriate cell types to pathophysiological culture conditions or NAFLD-inducer drugs. The physiologically healthy medium contained 5.5 mM glucose and 0.1 nM insulin, whereas pathologic media consisted of a high level of free fatty acid (FFA), insulin, and monosaccharides in line with the human *in vivo* NAFLD environment (Zhang et al., 2014). Based on current research, NAFLD models were mainly established with PHH cell type (Table 3).

**TABLE 3:** Selected 3D NAFLD models established from different cell types.

Cell type	Culture paradigm	Exposure duration	Endpoints/outcomes	References
PHHs	Spheroid mono-culture	48 h	Hepatic lipid accumulation	Bell et al. (2016)
PHHs	Spheroid mono-culture	21 days	Hepatic lipid accumulation	Kozyra et al. (2018)
			Transcriptional changes in genes associated with hepatic gluconeogenesis, glycolysis, and lipogenesis	
PHHs	Liver-on-a-chip mono-culture	14 days	Hepatic lipid accumulation	Kostrzewski et al. (2017)
			Transcriptional changes in genes associated with insulin resistance and lipid metabolism	
			Increased inflammatory and fibrotic markers	
PHHs	MPCC co-culture	18 days	Hepatic lipid accumulation	Davidson et al. (2016)
			Decreased sensitivity to insulin-mediated inhibition of glucose output and dysregulated insulin signaling	
PHHs	Liver-on-a-chipco-culture	10 days	Hepatic lipid accumulation	Feaver et al. (2016)
			Decreased sensitivity to insulin-mediated inhibition of glucose output and dysregulated insulin signaling	
			Increased oxidative stress and apoptosis	
			Increased inflammatory analyte secretion and fibrogenic activation markers	
HepG2	Gut–liver-on-a-chip co-culture	24 h	Accumulated chylomicrons in HepG2	Lee & Sung (2018)
hiPSC-HLCs; hESC-HLCs	Organoid tri-lineage	7 days	Hepatic lipid accumulation	Ouchi et al. (2019)
			Increased inflammatory cytokines	
			Increased collagen production	
			Increased organoid stiffness	

PHHs, primary human hepatocytes; MPCC, micropatterned co-culture; hESCs, human embryonic stem cells; hiPSCs, human induced pluripotent stem cells; HLCs, hepatocyte-like cells.

**FIGURE 2:**
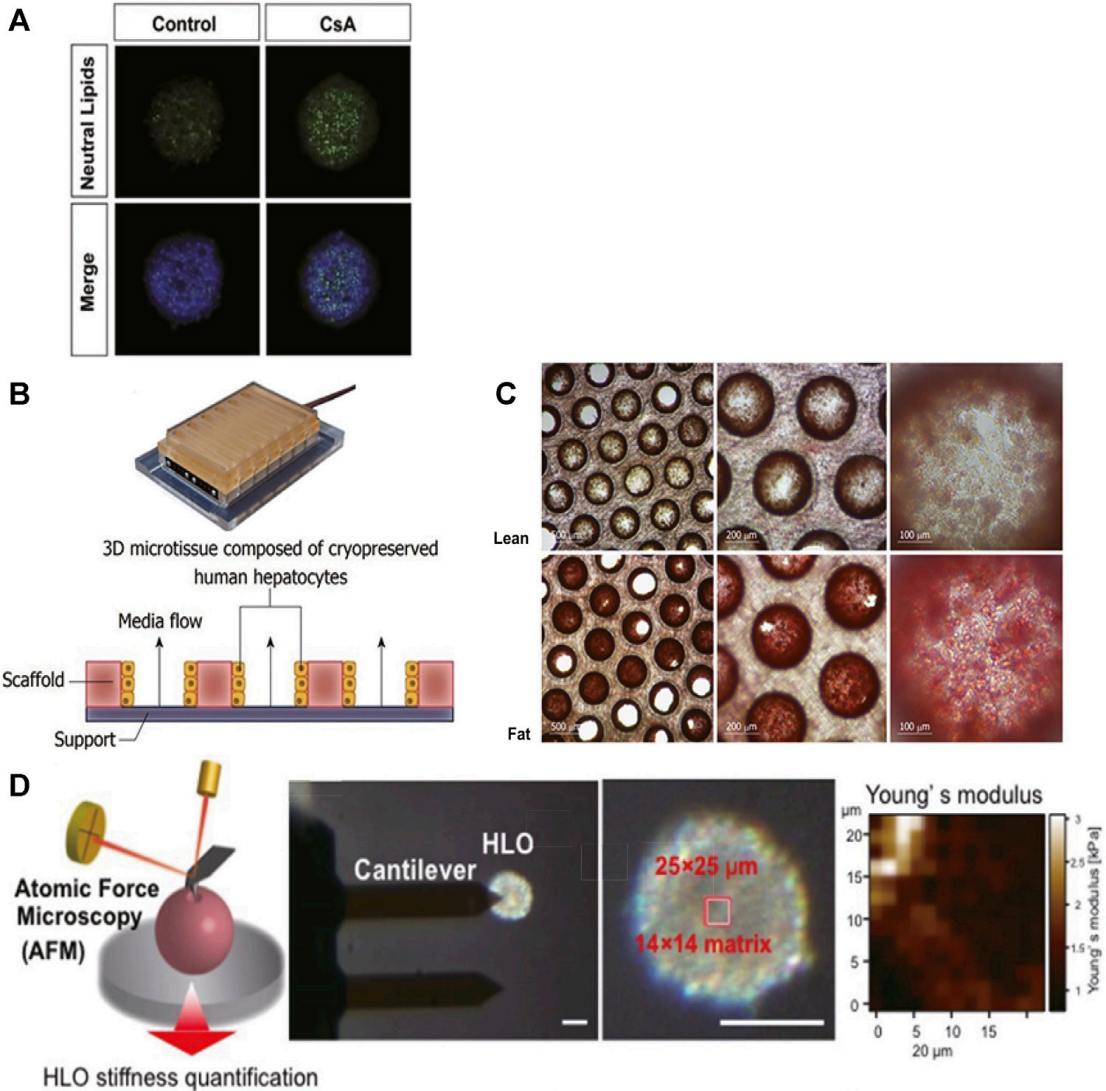
3D cell culture paradigms support disease modeling of NAFLD. **(A)** Representative pictures of lipid accumulated in PHH spheroids upon exposure to cyclosporine A (30 μM) for 48 h; scale bars, 100 μm. **(B)** Illustration of the PHH liver-on-a-chip model. **(C)** Intracellular fat accumulation in the liver-on-a-chip model under fat condition (Lower) was noted after Oil Red O staining. **(D)** Schematic demonstration of stiffness measurement by AFM. The top region of each single organoid (14 × 14 matrix in a 25 × 25 μm square) was scanned with an AFM cantilever. *Scale bar, 100 μm.* Young’s modulus on each scanned spot is shown in the heatmap. **(A)** Modified with permission from Bell et al. (2016). **(B,C)** Modified with permission from Kostrzewski et al. (2017). **(D)** Modified with permission from Ouchi et al. (2019).

Mono-cultured PHH spheroids and liver-on-a-chip models have been reported with successfully mimicked lipid accumulation, steatosis, insulin resistance, transcriptional change in relevant genes, and expression of inflammatory and fibrotic markers. Bell and colleagues demonstrated that steatosis was induced in PHH spheroids by exposure to probe drug cyclosporine A (30 μM) for 48 h, indicating the PHH spheroid was a promising model of drug-induced steatosis (Figure 2A) (Bell et al., 2016). Kozyra and colleagues further illustrated that transcriptional change related to steatosis and insulin resistance can be induced in PHH spheroids with pathophysiological FFA, monosaccharides, and insulin. After 14 days of exposure, increased expression of genes associated with insulin resistance includes phosphoenolpyruvate carboxykinase 1 (PCK1) mRNA, pyruvate dehydrogenase lipoamide kinase isozyme 4 (PDK4) mRNA, glucose-6-phosphatase mRNA, and fatty acid synthase mRNA, and decreased response of glycogen synthase kinase 3 (GSK3β) to insulin all indicated insulin resistance (Kozyra et al., 2018). In a liver-on-a-chip model, the microfluidic dynamic system allows constant exposure to FFA. Therefore, it can imitate the chronic conditions of fat accumulation in line with clinical patients. Kostrzewski and colleagues established a PHH liver-on-a-chip model in which the PHHs were exposed to fat culture conditions (600 μmol/L FFA under constant flow) for 14 days (Figures 2B,C), and then metabolic, transcriptome, and phenotypic changes were measured. Lipid accumulation and numerous transcriptional changes in genes associated with insulin resistance and lipid metabolism were noted from day 7, including increased CYP2E1, insulin-like growth factor (IGFβ1), PDK4, and CYP7A1 expressions. Meanwhile, the expression of inflammatory and fibrotic markers was noted, including interleukin-8 (IL-8), migration inhibitory factors, fibrinogen, and tissue inhibitor of metalloproteinase-1 (TIMP-1) (Kostrzewski et al., 2017).

In contrast, 3D co-culture models can be used to imitate a more complex phenotype of NASH, such as liver fibrosis, gut–liver interaction, and chronic hyperglycemia exposure. For that, PHHs can be co-cultured with human KCs, HSCs, 3T3-J2 murine embryonic fibroblasts, or gastrointestinal cells together, as HSCs are the main collagen-producing cells during hepatic parenchymal injury, KCs play an essential role in inflammation, and 3T3-J2 murine embryonic fibroblasts help maintain PHH function in MPCC models. Davidson and colleagues developed a PHH MPCC model to mimic hyperglycemia-induced lipid accumulation. After 18 days of chronic exposure to hyperglycemia (25 mM), MPCC showed more insulin-accumulated vesicles (∼2.1 fold) than MPCC exposed to normoglycemic media (5 mM). Impaired inhibitory effect of insulin (10 and 100 nM) on glucose output was observed in hyperglycemic MPCCs (detected glucose output reduced ∼70%) when compared with normoglycemic MPCCs (completely undetectable). The altered nuclear-to-cytoplasmic ratio (N:C) of forkhead box O1 (FOXO1) in hyperglycemic MPCCs relative to normoglycemic MPCCs also implied abnormal influence of insulin on translocation of FOXO1 as seen in insulin-resistance patients (Davidson et al., 2016). The liver-on-a-chip co-culture model represents similar disease progression to the MPCC model as well. Feaver et al. established liver-on-a-chip co-culture models that incorporated sinusoidal flow with PHHs, HSCs, and human macrophages. Upon 10 days of exposure to a lipotoxic environment consisting of elevated glucose (25 mM), FFA (110 μM), and insulin (6,900 pM) levels, the NASH phenotype was observed to be consistent with clinical NASH biopsies, manifesting as activated HSCs, insulin resistance (decreased sensitivity to insulin-mediated inhibition of glucose output and upregulated PCK1), mitochondrial dysfunction, increased oxidative stress (1.4-fold increased release of ALT and 2.6-fold increased CK-18), elevated inflammatory signaling (IL-8, IL-6, CXCL10, YKL40, upregulated toll-like receptors, and STATs), and increased fibrotic markers (TGF-β and osteopontin) as well as lipid accumulation (Feaver et al., 2016). Moreover, co-culturing enterocytes and hepatic cell types together has the potential to recapitulate the whole process from absorption to metabolism of digestive lipids in NAFLD patients, while conventional models only focused on hepatic response to excessive FFA. Lee and Sung developed the first gut–liver-on-a-chip model, in which Caco-2 enterocytes and HepG2 cells were incorporated in one chip. Upon treating the Caco-2 enterocytes with FFA for 24 h, lipid accumulation in HepG2 cells was observed. The role of the pro-inflammatory cytokine TNF-α in the gut absorption permeability of lipids was further demonstrated in this model, indicating its application in addressing the multi-tissue etiology of NAFLD and exploring novel drug targets outside the liver (Lee and Sung, 2018).

Organoids derived from hiPSC/hESC-HLCs co-cultured with HSC- and KC-like cells were applied in this field as well. Ouchi and colleagues reported the simultaneous differentiation of hiPSCs/hESCs into tri-lineage liver organoids containing HLCs and HSC- and KC-like cells. Upon 5 days of persistent exposure to FFA (oleic acid), hiPSC/hESC-HLCs accumulated intracellular lipids; enlarged, HSC-like cells upregulated collagen production (increased vimentin, α-smooth muscle actin, P3NP); and KC-like cells released pro-inflammatory cytokines (TNF-α, IL-6, and IL-8), recapitulating three key processes in NASH. Interestingly, organoid stiffness was measured by atomic force microscopy, and its increase likely reflected the severity of fibrosis (Figure 2D). Though the inter-batch variation in these organoids was reported in this experiment (Ouchi et al., 2019), it was proposed that stem cell–derived HLCs from patients would allow their specific NAFLD mechanisms to be assessed and benefit personalized medicine. For instance, patients with gene polymorphisms in patatin-like phospholipase-3 (PNPLA3) tend to suffer from high-grade NAFLD (Romeo et al., 2008), and stem cell–derived HLCs will allow the role of mutated gene in NAFLD to be studied. Meanwhile, advanced gene editing technology has the potential in not only revising diseased hiPSC-HLC organoids but also generating diseased hiPSC-HLC organoids with specific gene modulation as well (Alves-Bezerra et al., 2019; Cai et al., 2016). By contrast, these techniques might not be performed with patients’ PHHs, which do not have sufficient long-term stability under *in vitro* culture conditions for gene editing and organoid generation.

Collectively, human 3D hepatic *in vitro* NAFLD models were mainly derived from PHH cell type as it possesses *in vivo–*like metabolism of lipid and glucose and inducible insulin-resistance response. Importantly, they can be established by either mono-culture or co-culture, depending on the phenotypes to be imitated. Compared with the mono-culture model, the co-culture model is suitable for imitating more complex disease phenotypes. By incorporating 3T3-J2 murine embryonic fibroblasts, HSCs, KCs, or gastrointestinal cells with PHHs together, MPCC, organ-chip, and organoid co-culture models have been established, which successfully imitated liver fibrosis, gut–liver interaction, and chronic hyperglycemia exposure. In addition to pathogenesis modeling, several anti-NAFLD drug compounds have been tested based on PHH models (Bell et al., 2016; Feaver et al., 2016; Kostrzewski et al., 2017; Kozyra et al., 2018; Lee and Sung, 2018), indicating the PHH model can be a solution to accumulate sufficient preclinical evidence for clinical drug development. Furthermore, though current inter-batch variation in hiPSC-HLC–derived organoids was non-negligible, stem cell–derived HLCs would benefit personalized *in vitro* NAFLD modeling, which could be the significant advancement in the future modeling paradigm (Romeo et al., 2008; Alves-Bezerra et al., 2019; Ouchi et al., 2019).

#### Viral Hepatitis

**FIGURE 3:**
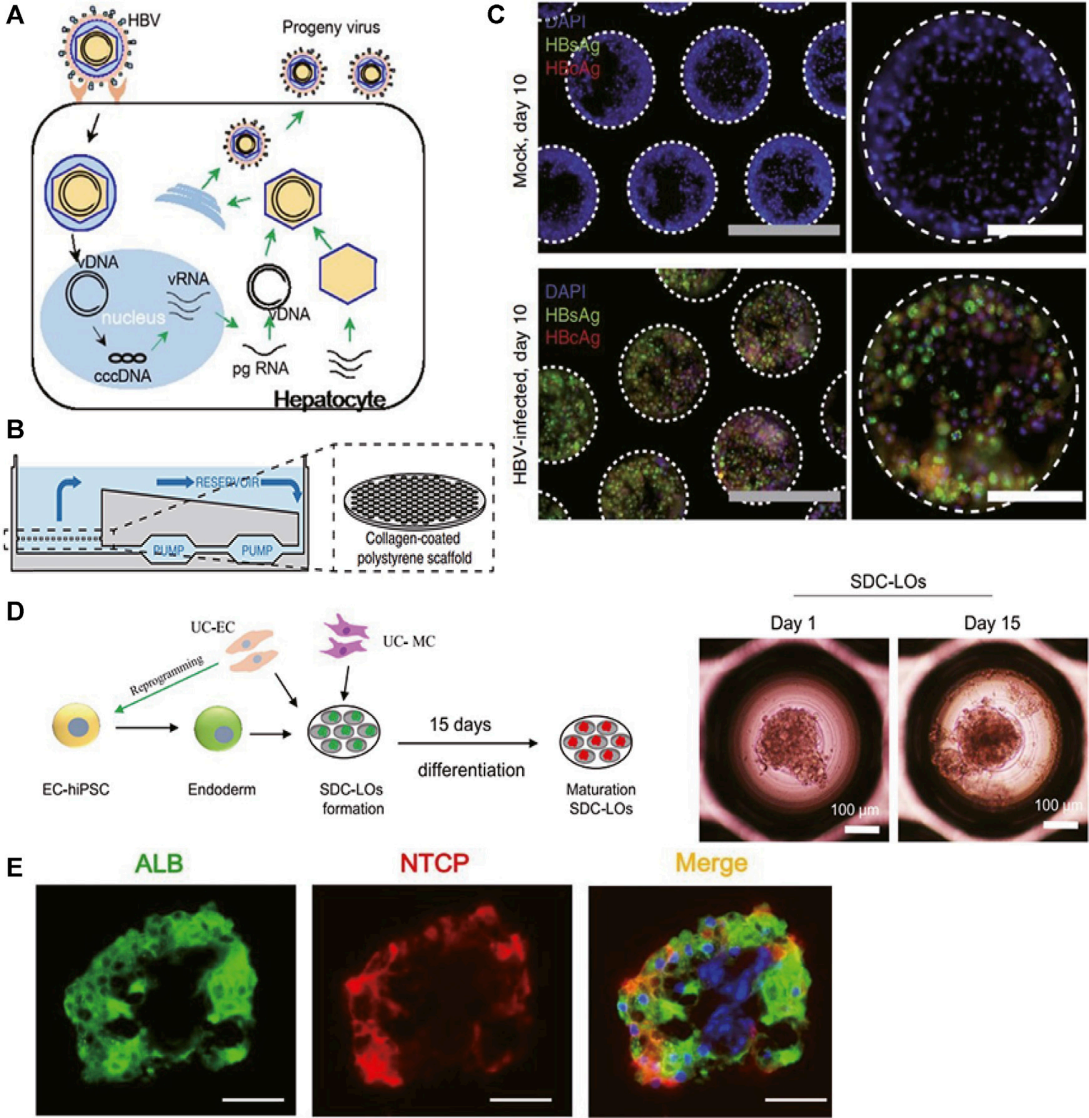
Selected representative 3D models of viral hepatitis B. **(A)** Schematic representation of the HBV life cycle in infected hepatocytes. **(B)** Illustration of the PHH liver-on-a-chip model. Microfluidic recirculation was driven by a micro-pump and went through collagen-coated polystyrene scaffolds seeded with PHHs. **(C)** Immunofluorescence of HBV viral antigens (HBsAg and HBcAg) 10 days following infection of the PHH liver-on-a-chip model with patient-derived HBV (100 GE/cell). **(D)** (Left) Schematic representation of the protocol for liver organoid generation and differentiation from single donor umbilical cord (UC)-derived hiPSCs, HUVECs, and mesenchymal cells (MCs). (Right) Morphology of single donor cell–derived liver organoids (SDC-LOs) on days 1 and 15. *Scale bars, 100 μm*. **(E)** Immunofluorescence staining of ALB and NTCP in differentiated SDC-LOs. Green, ALB; red, NTCP. *Scale bars, 50 μm*. **(A)** Modified with permission from Yan et al. (2012). **(B,C)** Modified with permission from Ortega-Prieto et al. (2018). **(D,E)** Modified with permission from Nie et al. (2018).

Viral hepatitis mostly results from hepatotropic virus A, B, C, D, and E infections through the fecal–oral, parenteral, perinatal, or sexual route. Among them, chronic HBV and HCV infections particularly lead to hepatic parenchymal damage including liver cirrhosis and HCC, and they are responsible for about 90% of viral hepatitis–leading annual deaths (Wiktor and Hutin, 2016). Current viral hepatitis modeling mainly focuses on HBV infection. HBV genome is a small (3.2 kb) and partially double-strand DNA, which persists as a covalently closed, circular (ccc) DNA episome (Figure 3A). This cccDNA is the transcriptional template for pregenomic (pg) RNA and subgenomic (sg) RNA species. Sodium-taurocholate co-transporting polypeptide (NTCP) was identified as one of the HBV entry receptors (Yan et al., 2012).

The goal in imitating HBV infection is to establish *in vitro* models that support productive infection and mimic virus–host interaction accurately, which will benefit the drug development targeting–specific viral life cycle and host responsiveness. PHH 2D mono-culture was thought to lose its permissiveness to HBV infection due to down-regulation of NTCP expression after isolation and subsequent culture (Yan et al., 2012). Therefore, Shlomai and colleagues co-cultured PHHs and stromal fibroblasts (3T3-J2 murine embryonic fibroblasts) in the MPCC model to mimic HBV infection. After exposure to HBV infectious serum at multiplicity of infection (MOI) of 300 HBV GE/cell, this MPCC model stably expressed NTCP receptors over 14 days and supported productive HBV infection over 21 days, reflected by robust viral gene expression including HBV surface antigen (HBsAg) secretion, HBV transcript mRNA production, HBV core antigen (HBcAg) expression, and the presence of high viral cccDNA copies. Innate immune response was observed as upregulated antiviral interferon-stimulated genes (ISGs) including interferon (IFN)-α and IFN-β, which were mainly induced after 7 days of infection (Shlomai et al., 2014).

Ortega-Prieto et al. mono-cultured PHHs in the liver-on-a-chip model (Figure 3B) to build a more natural host cell environment resembling liver sinusoids. HBcAg, HBsAg (Figure 3C), cccDNA, and HBV replication intermediates (sgRNA, pgRNA) were noted as well when treated with 100 GE/cell patient-derived HBV. Furthermore, both IFN-α and IFN-β were suppressed to 10% of the levels observed in mock-infected cultures, suggesting an active role of HBV in suppressing innate immune activation. Similar to the findings in the sera of HBV-infected patients, significantly elevated protein levels of IL-8, macrophage-inflammatory protein (MIP)-3α, SerpinE1, and monocyte chemotactic protein-1 (MCP-1) were observed in HBV-infected liver-on-a-chip models. Furthermore, to identify the role of non-parenchymal cells in HBV infection, Ortega-Prieto et al. co-cultured PHHs and KCs together in the above model. Surprisingly, except for the elevated level of C-reaction protein (CRP), virological characteristics of HBV infection including secreted levels of HBsAg, hepatitis B e antigen (HBeAg), pgRNA, and sgRNA were identical in mono-culture and co-culture, and KC-specific cytokines IL-6 and TNF-α were not secreted as well, indicating that HBV evaded the immunosurveillance function of liver-resident macrophages (Ortega-Prieto et al., 2018).

As illustrated in Figure 3D, Nie and colleagues co-cultured hiPSC-derived endoderm cells, human bone marrow mesenchymal cells, and human umbilical vein endothelial cells (HUVECs) together to establish a liver organoid that exhibited stronger hepatic functions and more susceptibility to HBV infection than hiPSC-HLC mono-culture organoids. Significantly increased expression levels of the NTCP (Figure 3E), viral pgRNA, cccDNA, and supernatant viral DNA were observed in organoids from the sixth day post-infection and can be maintained to at least 20 days post-infection. Meanwhile, infectious progeny viruses could still be produced from infected organoids at 20 days post-infection, suggesting this model could serve as a long-term *in vitro* infection model. Furthermore, the expression of HBcAg and known infection-promoting factors including glypican 5 (GPC5), peroxisome proliferator–activated receptor alpha (PPARA), and CCAAT/enhancer-binding protein alpha (CEBPA) was detected. Viral dose-dependent hepatic dysfunction with down-regulation of hepatic gene expression, induced release of early acute liver failure markers, and altered hepatic ultrastructure were noted, indicating this organoid could be a robust infection model representing viral host response (Nie et al., 2018).

As concluded in Table 4, PHHs and hiPSC-HLCs were proved to be suitable for modeling HBV infection as they recapitulate viral replicating cycles and viral–host interaction. Though the HepG2 cell line was susceptible to HBV infection as well (Sells et al., 1987; Gripon et al., 2002; Lucifora et al., 2010), concerning the virus–host interaction, it is not a suitable cell system since no ISG induction was observed after viral exposure, which was probably caused by defective HBV sensors or key transducers in this sensing pathway (Shlomai et al., 2014). Moreover, divergence in host response was observed in PHHs from different donors, implying the HLC cell model derived from hiPSCs or hASCs may benefit personalized treatment. Regarding the culture paradigm, in previous 2D models, PHHs and hiPSC-HLCs both supported HBV and HCV infections, which cannot sustain in the long term due to rapid loss of hepatic function (Roelandt et al., 2012; Schwartz et al., 2012; Shlomai et al., 2014). MPCC, liver-on-a-chip, and organoid models are more susceptible to HBV infection than the spheroid model since the central cells of spheroids are hard to get access to virus, and the infection was proved to be more effective if performed upon seeding rather than when cells had aggregated into spheroids (Bell et al., 2016). Besides, MPCC, liver-on-a-chip, and organoid models all support antiviral drug testing, such as HBV entry inhibitors, anti-HBV nucleoside, HBV reverse transcriptase inhibitors, and IFNα, validating these models as preclinical platforms for evaluating the novel treatment strategy (Shlomai et al., 2014; Nie et al., 2018; Ortega-Prieto et al., 2018).

**TABLE 4:** Selected 3D viral hepatitis models established from different cell types.

Cell type	Culture paradigm	Culture period	Endpoints	Application	References
PHHs	MPCC co-culture	21 days	Expression of NTCP receptors	Disease modeling: HBV infection	Shlomai et al. (2014)
			Expression of HBsAg and HBcAg		
			Expression of HBV DNA and replication intermediates		
			Induced ISG gene expression		
PHHs	Liver-on-a-chip mono-culture	22 days	Expression of HBsAg and HBcAg	Disease modeling: HBV infection	Ortega-Prieto et al. (2018)
			Expression of HBV DNA and replication intermediates		
			Suppressed baseline innate immune activation		
			Similar chemokine and cytokine responses as seen in HBV-infected patients		
hiPSC-HLCs	Organoid co-culture	42 days	Expression of NTCP receptors	Disease modeling: HBV infection	Nie et al. (2018)
			Expression of HBV DNA and replication intermediates		
			Expression of HBcAg and known infection-promoting factors		
			Viral dose-dependent hepatic dysfunction		

PHHs, primary human hepatocytes; hiPSCs, human induced pluripotent stem cells; HLCs, hepatocyte-like cells; NTCP, sodium-taurocholate co-transporting polypeptide; ISG, interferon-stimulated gene; MPCC, micropatterned co-culture; HBcAg, HBV core antigen.

#### Monogenetic Liver Disease

Resulting from mutation in one single gene, monogenic liver diseases include a group of hepatic functional disorders and can be divided into two types: one is manifesting as predominant hepatic parenchymal damage and the other is liver-based genetic disorders with an architecturally near-normal liver. This review focuses on the former since disease manifestations of this type are more likely to be fully recapitulated in hepatic models. The representative diseases are A1AT deficiency, Wilson’s disease, and Wolman’s disease. With mutation in the *SERPINA1* gene, A1AT deficiency results in decreased normal A1AT which protects the hepatic and pulmonary parenchyma from proteolytic damage of neutrophil elastase, predisposing to chronic hepatic and obstructive pulmonary diseases. The accumulation of misfolded A1AT in the endoplasmic reticulum (ER) of hepatocytes also triggers ER stress and apoptosis (Greene et al., 2016). With mutation in the *ATP7B* gene, Wilson’s disease results in inadequate functional copper-transporting ATPase 2, which transports copper out of the liver and prevents intracellular copper accumulation. Copper overload causes hepatocyte death and uncontrolled release of copper in the circulation (Ala et al., 2007). With mutation in the lysosomal acid lipase (LAL) gene, Wolman’s disease results in accumulation of lipids due to lack of the LAL enzyme and abnormal enzymatic breakdown of triglycerides, accompanied by hepatomegaly and lethal steatohepatitis (Pericleous et al., 2017). Though originated in different genetic backgrounds, underlying mechanisms of all these disorders involve continuous inflammation, parenchymal necrosis, abnormal hepatocyte regeneration, and ultimately the development of hepatic failure.

**FIGURE 4:**
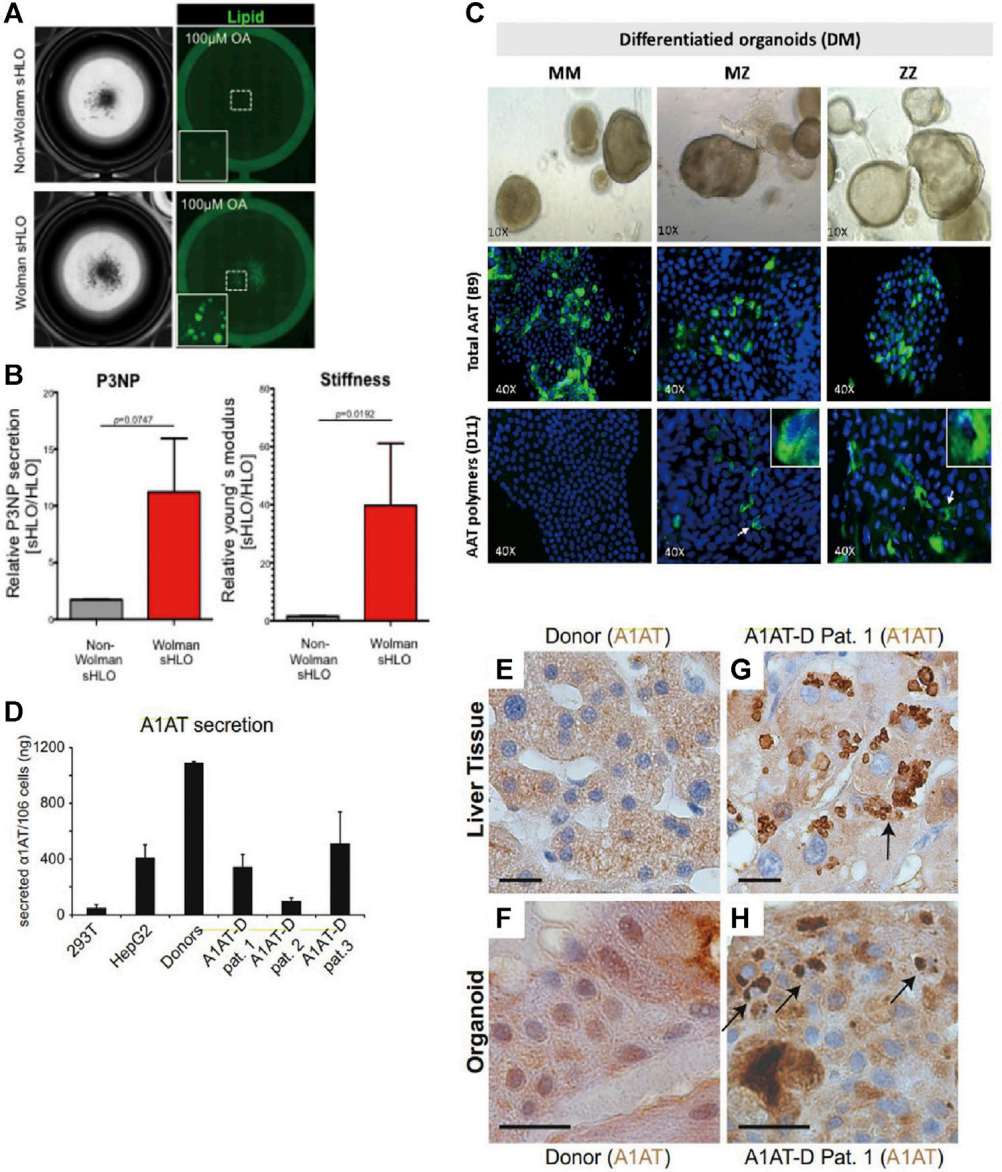
Selected representative 3D models of monogenic diseases. **(A)** Representative bright-field and lipid-fluorescent images of organoids from Wolman disease patients and non-Wolman donors after oleic acid treatment. **(B)** (Left) Comparison of the P3NP secretion level of non-Wolman and Wolman human liver organoids (HLOs). (Right) Comparison of Young’s modulus of non-Wolman and Wolman organoids, *n* = 7. **(C)** Differentiated liver organoids from MM, MZ, and ZZ patients. Specific detection of total AAT protein with anti-AAT-B9 and AAT polymers with anti-AAT-D11 is shown in green fluorescence. **(D)** ELISA measurement of A1AT secretion in supernatants from healthy donor and patient organoids after 11 days of differentiation. **(E–H)** Immunohistochemistry for A1AT on liver tissue **(E,G)** and liver-derived organoids from a healthy donor **(F)** and a representative A1AT deficiency patient **(H)**. Black arrows indicate A1AT proteins aggregated in patient-derived liver tissue **(G)** and organoids **(H)**. *Scale bars, 20 mm.* All results are represented as mean ± SEM. Figure modified with permission from Ouchi et al. (2019), Gomez-Mariano et al. (2020), and Huch et al. (2015).

For Wolman’s disease, Ouchi and colleagues derived organoids from the patient’s hiPSC-HLCs. Upon exposure to oleic acid, prominent lipid accumulation in Wolman’s disease organoids compared with normal hiPSC-derived organoids was noted (Figure 4A), and it was rescued after exposure to recombinant LAL protein. 11.22-fold fibrotic marker P3NP (2.8-fold in normal hiPSC-derived organoids) and significantly increased stiffness further suggest Wolman’s disease organoids exhibit more aggressive fibrosis phenotypes (Figure 4B) as seen in clinical patients (Ouchi et al., 2019).

Huch and colleagues developed the A1AT deficiency organoid model via using hASCs (EpCAM+) obtained from patient biopsies with homozygous (*ZZ*) A1AT mutants. After 22 days of expansion and differentiation, around 67% reduced A1AT secretion in the culture supernatant accompanied by reduced elastase inhibition and sign of increased ER stress (increased phosphorylation of eIF2a) was noted, indicating successful imitation of hallmarks of A1AT deficiency *in vitro* (Figure 4D). Meanwhile, similar to those found in the original patient biopsies, aggregated A1AT proteins were observed after 11 days of differentiation under immunohistochemistry staining (Figures 4E–H) (Huch et al., 2015). Furthermore, to study the genotype-specific feature in A1AT deficiency, Gomez-Mariano and colleagues took 35 days to establish organoids from liver biopsies of patients with the homozygous (*ZZ*) variant, the heterozygous (*MZ*) variant, and normal (*MM*) genotypes of A1AT. In contrast to *MM* phenotypes, a lower transcription level of the *SERPINA1* gene was found in *ZZ* organoids. Upon immunohistostaining and quantification of cells positive for the A1AT polymer, 5%, 10% of cells in *MZ*, *ZZ* organoids exhibited polymer accumulation, respectively (Figure 4C). Meanwhile, western blot revealed that the monomeric A1AT protein was detected in the extracellular medium and cell extracts of *MM*, *MZ*, but not *ZZ* organoids, while the largest amount of insoluble A1AT was present in *ZZ* compared to *MZ* or *MM* organoids. The above findings reflecting genotype-specific features were also observed in patients (Gomez-Mariano et al., 2020). Since there is no consensus guideline for these monogenic diseases and the current ameliorative strategies are expensive, these organoid models were further exploited to investigate the therapeutic potential of drug compounds to reverse the phenotype of lipid accumulation and stiffness (Ouchi et al., 2019), or induce increased expression of full length or short transcripts of A1AT (Gomez-Mariano et al., 2020).

In summary**,** current 3D cell models of monogenic diseases are mainly developed via using the patient-derived cell sample, including hASCs isolated from liver biopsy and hiPSCs (Table 5). These studies are proof-of-principle that liver organoids recapitulated key features of the disease *in vitro*. Development of the model derived from the patient’s materials is valuable since the mutation of alleles in one single gene is diverse. Together with environmental and epigenetic factors, they all may lead to different clinical presentation of diseases, while cell samples from patients fill this research gap and recapitulate the genetic background of individuals. Combined with gene editing, specific genetic therapy can be designed to benefit personalized treatment based on these patient-derived organoid models.

**TABLE 5:** Selected 3D monogenic liver disease models established from different cell types.

Cell type	Culture paradigm	Culture period	Endpoints	Application	References
Patient’s hASC-HLCs	Organoid mono-lineage	22 days	Decreased A1AT secretion	Disease modeling: A1AT deficiency	Huch et al. (2015)
			Polymerized A1AT intracellular aggregation		
			Reduced ability to block elastase activity		
			Increased ER stress		
Patient’s hASC-HLCs	Organoid mono-lineage	35 days	Decreased *SERPINA1* transcriptional expression	Disease modeling: A1AT deficiency	Gomez-Mariano et al. (2020)
			Decreased normal A1AT secretion		
			Polymerized A1AT intracellular aggregation		
Patient’s hiPSC-HLCs	Organoid mono-lineage	25 days	Hepatic lipid accumulation	Disease modeling: Wolman’s disease	Ouchi et al. (2019)
			Increased fibrotic marker		
			Increased stiffness		

hASCs, human adult stem cells; HLCs, hepatocyte-like cells; hiPSCs, human induced pluripotent stem cells; A1AT, alpha-1 antitrypsin.

### Hepatocyte Transplantation

Liver transplantation is the accepted treatment for patients with end-stage liver failure, while shortage of liver donors and lifelong need for immunosuppression are critical challenges for accepting allogenic liver transplantation. Meanwhile, hepatocyte transplantation as an alternative to liver transplantation is also impeded by shortage and the expanding challenge of PHHs (Iansante et al., 2018). Under these circumstances, generating *in vivo* liver tissue like a 3D hepatic model *in vitro* with pluripotent stem cell–derived HLCs becomes an important issue, which enables homologous hepatocyte transplantation with the potential of limiting immune rejection. For transplantation, maturity and complete function of established liver tissue are significant. Current studies co-cultured hiPSCs with various types of supporting non-parenchymal cells to attain a higher differentiation yield and to improve HLC functions both *in vitro* and *in vivo*. Organoid models established from pluripotent stem cell–derived HLCs or using 3D bioprinting technology are promising to provide possible solutions (Takebe et al., 2013; Hu et al., 2018; Pettinato et al., 2019; Yang et al., 2021). The idea of applying hiPSC/liver progenitor-HLC organoids in regenerative medicine is based on the natural regeneration of the liver. Liver progenitor cell–driven regeneration was reported upon liver injury. During this process, preexisting hepatocytes or biliary epithelial cells dedifferentiated into liver progenitor cells, followed by proliferation and differentiation of progenitor cells into hepatocytes to rapidly restore tissue mass (Yanger et al., 2014; So et al., 2020).

Takebe and colleagues co-cultured hiPSC-hepatic endodermal cells with human mesenchymal stem cells (MSCs) and HUVECs to generate an organoid that developed into hepatic tissue *in vivo* upon transplantation into NOD/SCID mice. The three cell lineages spontaneously formed a vascular-like endothelial network and organized into 3D organoid tissue *in vitro*, resembling the *in vivo* liver bud through immunohistochemistry staining and gene expression analysis. The vasculature in organoids successfully connected to the host vessels within 48 h, stimulating its maturation into adult liver-like tissue *in vivo.* Liver-specific function including albumin secretion and human-specific ketoprofen and debrisoquine metabolism were reported in mice after transplantation. Moreover, mesenteric transplantation of the liver bud rescued drug-induced acute liver failure in the TK/NOD mouse model, suggesting the use of hiPSC-HLC organoids as a novel source for liver transplantation (Takebe et al., 2013). Yang et al. constructed the liver organoid derived from HepaRG and bioink following specific 3D bioprinting procedures and proved its liver functions *in vitro* and *in vivo*. In this study, the HepaRG cell line was selected for its dual potential to differentiate into functional hepatocytes and cholangiocytes. Hepatic functions including albumin secretion, drug metabolism, and glycogen storage were detected after 7 days of differentiation of HepaRG *in vitro*. Upon transplanted into the *Fah* ^*−/−*^ *Rag2* ^*−/−*^ mouse model of liver injury, 3DP-HOs displayed functional vasculature, increased synthesis of liver-specific proteins including human albumin, A1AT, and factors VII and IX, and exhibited human-specific debrisoquine metabolism. Significantly, transplantation of 3DP-HOs prolonged the cumulative survival of mice from 32 to 56 days and decreased the weight loss, indicating that 3D bioprinting could be used to generate human liver tissues as the alternative transplantation donors. Interestingly, the authors conducted a pilot study investigating different cell types in 3D bioprinting and indicated that the survival rate of PHHs after printing was too low (less than 10%), and the *in vitro* liver functions of HepG2 cell lines were also not satisfactory. Among all tested cell types, the HepaRG cell line is the most appropriate alternative to PHHs because it possesses differentiation capability and comparable hepatic metabolic properties (Yang et al., 2021).

## Conclusion

Rapidly emerging *in vitro* human hepatic 3D models maintaining hepatic phenotypes and functions increase the predictability of DILI, allow for faithful metabolite profiling and disease imitating, and benefit exploration of idiosyncratic drug effects as well. PHHs, HepaRG, HepG2, hiPSC/hESC-HLCs, and hASC-HLCs were mostly involved in the above applications. Indicated by recent studies, different hepatic cell types possess unique genetic and protein expression profiles and show specific advantages in divergent research fields. Compared to HepaRG, HepG2, and all stem cell-HLCs, PHH cell type in the spheroid or MPCC model shows superior sensitivity, specificity, and convenience in drug hepatotoxicity screening. PHH MPCC models, supported by mouse embryonic 3T3 fibroblasts, show extended cell viability typically up to 6 weeks with sustained hepatic functions. Therefore, the PHH MPCC model particularly benefits metabolite profiling for drugs with low clearance. For idiosyncratic drug effect study, both PHHs and hiPSC/hASC-HLCs obtained from patients are recommended. In the field of disease modeling, co-culture models incorporating HSCs, KCs, or gastrointestinal cells with PHHs or hiPSC/hASC-HLCs together were suitable for imitating more complex disease phenotypes and show faithful disease-modulated immune response. Liver-on-a-chip, liver–gut-on-a-chip, and organoid models resemble human physiological structure and provide a more faithful *in vivo*–like environment. Current 3D models of monogenic diseases are organoid models derived from the patient-derived cell sample, including hASCs isolated from liver biopsy and hiPSCs. Healthy organoids derived from human stem cells/liver progenitor cells or combined with 3D printing materials are promising for liver transplantation.
